# Quick, “Imputation-free” meta-analysis with proxy-SNPs

**DOI:** 10.1186/1471-2105-13-231

**Published:** 2012-09-12

**Authors:** Christian Meesters, Markus Leber, Christine Herold, Marina Angisch, Manuel Mattheisen, Dmitriy Drichel, André Lacour, Tim Becker

**Affiliations:** 1 German Center for Neurodegenerative Diseases (DZNE), Bonn, Germany; 2 Institute for Medical Biometry, Informatics and Epidemiology, University of Bonn, Bonn, Germany; 3 AESKU.KIPP-Institute, Wendelsheim, Germany; 4 Department of Biostatistics, Harvard School of Public Health, Boston, Massachusetts, USA

## Abstract

**Background:**

Meta-analysis (MA) is widely used to pool genome-wide association studies (GWASes) in order to a) increase the power to detect strong or weak genotype effects or b) as a result verification method. As a consequence of differing SNP panels among genotyping chips, imputation is the method of choice within GWAS consortia to avoid losing too many SNPs in a MA. YAMAS (**Y**et **A**nother **M**eta **A**nalysis **S**oftware), however, enables cross-GWAS conclusions prior to finished and polished imputation runs, which eventually are time-consuming.

**Results:**

Here we present a fast method to avoid forfeiting SNPs present in only a subset of studies, without relying on imputation. This is accomplished by using reference linkage disequilibrium data from 1,000 Genomes/HapMap projects to find proxy-SNPs together with in-phase alleles for SNPs missing in at least one study. MA is conducted by combining association effect estimates of a SNP and those of its proxy-SNPs. Our algorithm is implemented in the MA software YAMAS. Association results from GWAS analysis applications can be used as input files for MA, tremendously speeding up MA compared to the conventional imputation approach. We show that our proxy algorithm is well-powered and yields valuable *ad hoc* results, possibly providing an incentive for follow-up studies. We propose our method as a quick screening step prior to imputation-based MA, as well as an additional main approach for studies without available reference data matching the ethnicities of study participants. As a proof of principle, we analyzed six dbGaP Type II Diabetes GWAS and found that the proxy algorithm clearly outperforms naïve MA on the *p*-value level: for 17 out of 23 we observe an improvement on the p-value level by a factor of more than two, and a maximum improvement by a factor of 2127.

**Conclusions:**

YAMAS is an efficient and fast meta-analysis program which offers various methods, including conventional MA as well as inserting proxy-SNPs for missing markers to avoid unnecessary power loss. MA with YAMAS can be readily conducted as YAMAS provides a generic parser for heterogeneous tabulated file formats within the GWAS field and avoids cumbersome setups. In this way, it supplements the meta-analysis process.

## Background

The ongoing GWAS era has led to 1,449 association findings for 237 complex traits by 6/2011 [1]. Typically small effect sizes, however, leave a large fraction of disease susceptibility unexplained, a phenomenon that has become famous as “the case of the missing heritability” [2-4]. Several potential explanations for the phenomenon were given, including over-estimation of the heritability, rare variants with larger effects, common variants with even smaller effects than observed so far, incomplete coverage of current GWAS marker panels, but also epigenetic effects [5] or interactions between genetic variants [6]. In order to address incomplete coverage and common variants with small effects, a most required method is an efficient combination of genome-wide association studies (GWAS) of the same objective. Meta-analysis (MA) is capable of improving the power of GWAS and to examine the heterogeneity between studies [7,8]. Available tools [9,10] for meta-analysis combine study data marker by marker. Markers which are not part of all included studies are underestimated in their contribution to the phenotype under investigation. Hence, such markers may be lost in further consideration, regardless of their actual disease association, simply due to misrepresented study power. Therefore, imputation is used to unify the available marker panels of GWAS and to avoid loss of SNPs that are present in one study but not in another. Conjunction of imputation and subsequent meta-analysis has become a standard technique for combination of GWAS data. Several imputation methods have been developed during the last years, including MaCH [11], IMPUTE [12,13], BIMBAM [14], BEAGLE [15], or EMINIM [16] which are widely used. However, imputation is a time-consuming step with high computational performance requirements, which can be conducted in acceptable time only on high performance computer clusters. Furthermore, the imputation accuracy varies greatly from SNP to SNP, which is difficult to take into account for meta-analysis, and, may result in a loss of power [17]. Hence, great care has to be taken by each research group contributing to a meta-analysis effort. Here, we present a MA-approach that directly operates on GWAS association results and that can be run within about 1 hour with our YAMAS (**Y**et **A**nother **M**eta **A**nalysis **S**oftware) software. In particular, it is possible to carry out a first analysis without the need to impute. The idea of our algorithm is that for SNPs that are present in one study but not in another, substitute proxy SNPs are defined using reference data from the HAPMAP [18] or 1,000 Genomes projects [19]. In this way, all SNPs that are present in at least one of the experimental marker panels can be analyzed. We evaluate the performance of the proxy algorithm with data sets that were simulated using realistic linkage disequilibrium patterns obtained from the 1,000 Genomes project. Moreover, we successfully applied our approach to Type II diabetes (T2D) GWAS data derived from the database of Genotypes and Phenotypes (dbGaP). [20].

## Results and discussion

### Simulation Study

We conducted a power study based on 1,000 Genomes [19] data (August 2010 release), in order to obtain realistic linkage disequilibrium (LD) patterns. We used the chromosome 22 data of the 288 individuals of European descent (CEU sample) as a “master” data set *M*. In general, we simulated series of new data sets *M*_*i*_ by random re-assignment of cases-control status, so that each *M*_*i*_ consisted of 144 cases and 144 controls. For each data set, we conducted a single-marker analysis on *M*_*i*_ and identified the smallest p-value $\text{min}P_{M_{i}}$ obtained for any of the SNPs in *M*_*i*_. In case $\text{min}P_{M_{i}}$ was smaller than 1×10^−6^, we kept the simulated data set for further analysis. We stopped the simulation process when 500 data sets with $\text{min}P_{M_{i}}<1\times 10^{-6}$ had been obtained. In order to compare the relative performance of the proxy algorithm and imputing we investigated to what degree $\text{min}P_{M_{i}}$ could be retrieved in a meta-analysis. For this purpose, *M*_*i*_ was split into two “studies” *A*_*i*_ and *B*_*i*_, each involving only 144 individuals. In addition, for *A*_*i*_ SNP information was kept only for SNPs from the Illumina^®^ Human660WQuad v1 panel, and for *B*_*i*_, SNP information was kept only for Affymetrix^®^ 6.0 chip content. Meta-analysis of *A*_*i*_ and *B*_*i*_ was then performed either using only the SNPs available in both panels (MA-intersection), or using the proxy algorithm (MA-proxy), or based on *A*_*i*_ and *B*_*i*_ imputed with the IMPUTE [13] software using the 1,000 Genomes data (MA-impute). The comparison of the three MA strategies was then based on their potential to recapture the association signal in the complete master sample. To meet this purpose, the p-values $\text{min}P_{\text{MA}-\text{intersection}}^{i,100\text{kb}}$, $\text{min}P_{\text{MA}-\text{proxy}}^{i,100\text{kb}}$ and $\text{min}P_{\text{MA}-\text{impute}}^{i,100\text{kb}}$ were defined by the smallest p-values obtained within the 100 kb window around the top signal $\text{min}P_{M_{i}}$ of the master file. The window size was chosen since LD typically extends about 50 kb [18]. Therefore, we investigated the regions 50 kb upstream and downstream of the top signal. Nominal “power” was then evaluated based on the *minP* values: we counted the fraction of data sets for which *minP* was smaller than *α* ∈{0.01,0.001,*..*,1×10^−6^}. For comparison, we will show also the power values that would be obtained when both studies *A* and *B* had been genotyped for all SNPs of the 1,000 Genomes panel (LIMIT). That reflects the upper limit of what the meta-analysis approaches could have reached. Adjustment for the number of SNPs within the 100 kb window was not performed. In particular, *minP*_*MA*−*proxy*_ and *minP*_*MA*−*impute*_ were treated equally, even though more SNPs were considered with imputation-based MA. Thus, MA-imputed was favored in a way by our power definition. However, in practice a significance level of 5×10^−8^ is the consensus to establish genome-wide significance, irrespective of the number of SNPs actually tested. Therefore, it seems to be appropriate not to adjust for the varying amount of SNPs tested when comparing different MA strategies.

We tried to mimic different scenarios of potential reference panels, an “ideal”, an “incomplete” and a “mismatched” one. First, the “ideal” reference panel represents a perfect and complete match of the haplotype distribution between the study and the reference data. The “ideal” reference panel consisted of exactly the same haplotypes as the master file. In practice, the reference data will contain only a fraction of the haplotypes that are actually present in the study population, simple because of the limited size of the reference samples. Therefore, our “incomplete” reference consisted only of a third of the haplotypes present in the master file, in order to mimic a real data analysis situation. Third, the “mismatched” reference consisted only of the haplotypes of African descent (YRI sample) and was included to investigate the robustness against poorly fitting reference panels.

### Running Time

Running time was evaluated on a cluster with 400 CPUs (2.4 Ghz, 2GB RAM). In Table 1, running time estimates (unix **time** command, real time) from analysis with YAMAS are listed. Analysis of the Type II Diabetes data (first line of Table 1, 6 studies) took 2 minutes and 5 seconds with the point-wise approach, and 92 minutes and 5 seconds with the proxy-algorithm, using a single CPU. In order to investigate running time in correlation with the number of studies, we increased the meta-analysis to 12 (24) studies by using each of the original dbGaP studies twice (four times). Running time of the point-wise algorithm is about proportional in the number of investigated studies: increasing the number of studies by a factor of 4 leads to an increase in running time from 2m5s to 6m56s, which corresponds to factor of 3.35. The running time of the proxy-algorithm grows only moderately with an increasing number of studies. For 24 studies, the running time is only about 8 minutes longer than with 6 studies. This is because most of the running time is needed for reading, indexing and storing the proxy reference file, which has to be done only once. We conclude that the proxy-algorithm can be run even with a large number of studies within less than two hours. In addition, several algorithms and data processing routines are parallelized, based upon the OpenMP project [21], such that the YAMAS running time can be further improved if required by the user.

**Table 1:** Running time estimates for different number of studies

**Numbers of studies**^**a**^	**MA-pointwise**^**b**^	**MA-proxy**^**c**^
6	2m5s	92m5s
12	3m42s	94m53s
24	6m56s	100m4s

^a^Number of studies examined by meta-analysis.

^b^Naive approach, real time in minutes (m) and seconds (s).

^c^Proxy algorithm, real time in minutes (m) and seconds (s).

We wish to contrast the running time of the proxy-algorithm of 92m5s (6 studies) to that of imputation. Imputation was carried out in chunks using IMPUTEv2 [13]. We used 300 CPUs such that we were able to analyze each in one go. The average running time of a chunk was 20 hours. Since 6 studies had to be analyzed, 300 CPUs run 120 hours, each. In addition, association analysis ran 5 hours on average per chromosome, using PLINK [10], i.e., 22 CPUs were needed for 30 hours, in addition. In total, but ignoring extra running time for merging of chunks, imputation and association testing took 300 · 20 · 6 + 22 · 5 · 6 = 36,600 CPU-hours which is 23,921 more than what is needed with the proxy-approach. When one assumes that ample CPUs are available to impute all chunks of one study in parallel, the running time the user actually has to wait is 20 · 6 + 5 · 6 = 150 hours, ignoring overhead that is need to check and format the data for association testing. Thus, even when a cluster with hundreds of CPUs is available, running time improves by a factor of almost one hundred with the proxy-algorithm (150*h*/92*m*5*s*=98).

### Results from Simulation Study

In Table 2 results from simulations under the null hypothesis are shown. None of the investigated methods exceeds the nominal level, of either *α* = 0.0 or *α*= 0.01, irrespective of the reference file that is chosen. In particular, there is no evidence for inflated type I error with the new proxy algorithm. This was also true when the *r*^2^-limit for a SNP and its proxy was relaxed from 0.80 to 0.50 (data not shown). We conclude that the proxy approach is a valid method. Of notice, all methods are too conservative when a random effects model is used. This is in concordance with a recent publication [22] in which is was shown that the random effects model tests an inappropriately strict null hypothesis.

**Table 2:** Empirical levels for different nominal alpha levels (0.01 and 0.05) using different reference data sets

**Strategy**^**a**^	**Reference**^**b**^	**Model**^**c**^	***α*** = **0.05**^**d**^	***α *= 0.01**^**e**^
MA-pointwise	-	fixed	0.047	0.009
	-	random	0.037	0.006
MA-proxy	ideal	fixed	0.046	0.009
		random	0.036	0.007
	incomplete	fixed	0.046	0.009
		random	0.036	0.006
	mismatched	fixed	0.047	0.009
		random	0.036	0.007
MA-impute	ideal	fixed	0.042	0.007
		random	0.035	0.006
	incomplete	fixed	0.041	0.007
		random	0.034	0.006
	mismatched	fixed	0.039	0.008
		random	0.032	0.006

^a^Enumeration using either the naive approach, proxy algorithm or imputation strategy.

^b^Reference data set used for proxy algorithm and imputation.

^c^Calculation of the allelic effects by the fixed effect or random effect model.

^d^Empirical levels for nominal *α* of 0.05 (average over all SNPs from chr 22 for 100 permutation replicates).

^e^Empirical levels for nominal *α* of 0.01.

**Figure 1:**
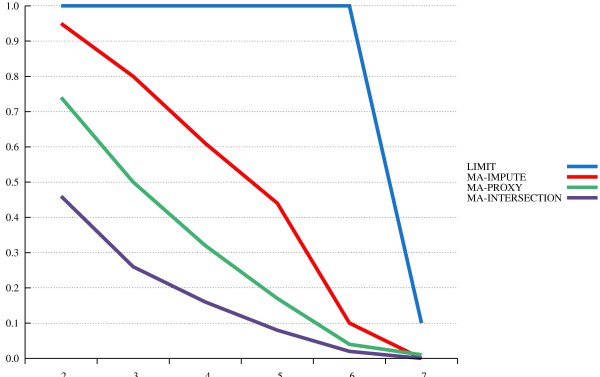
**MA with “ideal” reference panel.** Power levels are plotted over different nominal *α* levels (on the x-axis, with a negative logarithmic scale).

**Figure 2:**
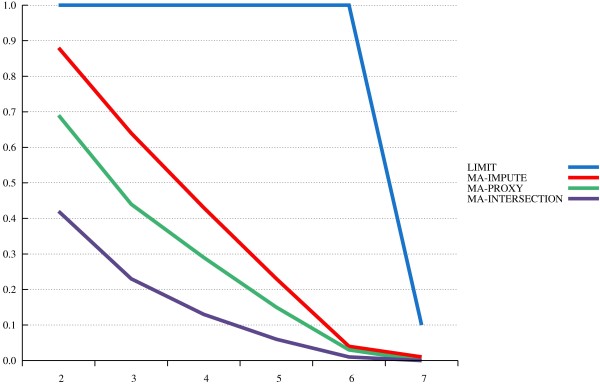
**MA with “incomplete” reference panel.** Power levels are plotted over different nominal *α* levels (on the x-axis, with a negative logarithmic scale).

**Figure 3:**
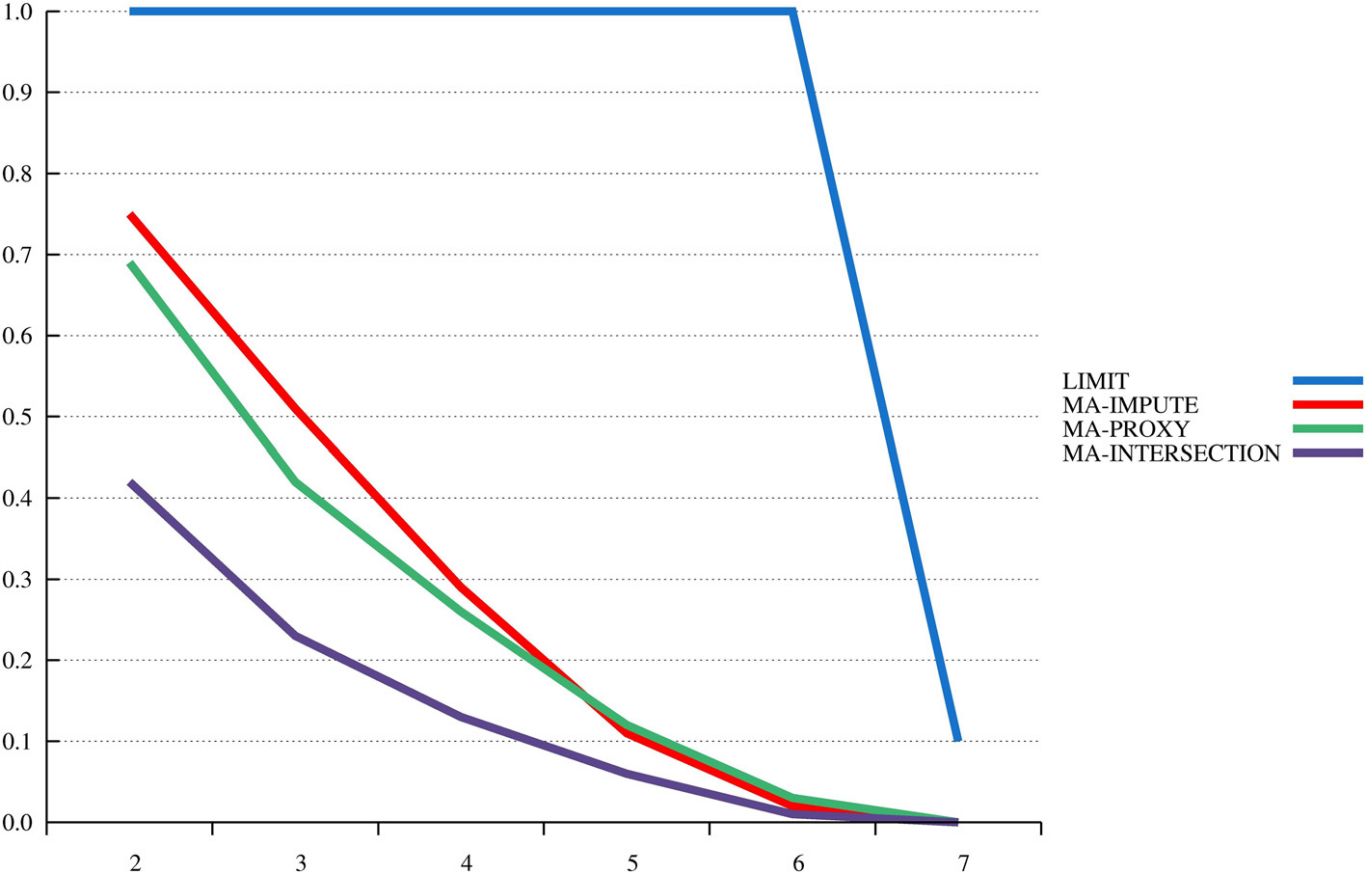
**MA with “mismatched” reference panel.** Power levels are plotted over different nominal *α* levels (on the x-axis, with a negative logarithmic scale).

The results from our power study are depicted in Figures 1, 2 and 3. The x-axis displays various *α*-levels on a logarithmic scale, moving from higher to lower levels. The y-axis displays power levels.

When an ideal reference panel is used (Figure 1), MA with imputing strongly outperforms naïve MA restricted to the joint marker panel (MA-intersection). Thus, imputing is highly recommendable. Nevertheless, the power level with MA-impute is considerably lower than power that can be achieved with a hypothetical sample that genotyped for all 1,000 Genomes SNPs (LIMIT). Thus, our simulation study also confirms the claim that imputing cannot replace complete genotyping or sequencing [23]. MA-proxy clearly outperforms MA-intersection, but is, as expected, less powerful than MA-impute. This is partly due to, first, the smaller marker panel that MA-proxy can analyze, and, second, that in case of incomplete LD the proxy marker will not necessarily reflect the true effect size.

In the presumably most realistic scenario (incomplete reference, Figure 2), we still see an impressive power gain with MA-impute when compared to MA-intersection. The performance of MA-proxy now comes much closer to that of MA-impute than with unrealistic “ideal” reference panel. We conclude that the proxy algorithm can yield valuable *ad hoc* results at an early analysis stage.

Thirdly, considering the mismatched reference panel (Figure 3), it is noteworthy, that MA-impute and MA-proxy still markedly outperform naïve MA. Obviously, even distant ethnical groups still share common LD patterns that can be useful in extending SNP information. Of note, there is no longer a measurable difference between the performance of MA-proxy and MA-impute. In summary, the difference between MA-impute and MA-proxy becomes smaller with reduced fit of the reference panel with the data. This is plausible: the imputing approach is the more sophisticated one, taking into account higher-order LD, whereas the proxy algorithm uses only pairwise LD information. Thus, the relative performance of the imputing approach will be the better the more closer its assumption “concordance of the study and reference haplotype set” is fulfilled. In contrast, the proxy algorithm uses a rougher metric, and, therefore, is more robust to peculiar mismatches in haplotype structure. As a consequence, the proxy algorithm can be recommended as an alternative main approach when a close-fitting reference panel is not available.

### Analysis of Type II Diabetes dbGaP Data

We examined the performance of the proxy approach on the basis of six Type II Diabetes GWAS studies that were available from dbGaP [20], cf. Table 3. The six GWAS studies belong to three different projects.

**Table 3:** Type II Diabetes dbGaP studies

**ID**	**Project**	**Study**	**Platform**	**SNPs**	**Individuals**	
1	Health Research Vanderbilt U^a^		Illumina^*®*^ Human660W-Quad v1	499,350	607	
2	Health Research Vanderbilt U^a^		Illumina^*®*^ Human1M-Duo v3	919,602	1,384	
3	Health Research Northwestern U^b^		Illumina^*®*^ Human660W-Quad v1	495,588	1,239	
4	Health Research Northwestern U^b^		Illumina^*®*^ Human1M-Duo v3	908,692	267	
5	GENEVA Diabetes Study	NHS^c^	Affymetrix^*®*^ Human SNP Array 6.0	764,678	3,435	
6	GENEVA Diabetes Study	HPFS^d^	Affymetrix^*®*^ Human SNP Array 6.0	787,213	2,606	

^a^Project Health Research - Vanderbilt University, Northwestern NUgene Project: Type 2 Diabetes, National Human Genome Research Institute (NHGRI).

^b^Project Health Research - Northwestern University, Northwestern NUgene Project: Type 2 Diabetes, National Human Genome Research Institute (NHGRI).

^c^Nurses health study.

^d^Health Professionals Follow-up Study.

There are two projects of the Northwestern NUgene Project Type 2 Diabetes from the National Human Genome Research Institute (NHGRI), each of which contributed 2 studies to our analysis. The “Project Health Research - Vanderbilt University” project provided two studies from different platforms. Data generated with the Illumina^®^ Human660W-Quad v1 chip comprised 607 individuals and 499,350 markers. Another fraction of patients were examined with the Illumina^®^ Human1M-Duo v3 array, for which 1384 individuals and 919,602 SNPs remained after quality control (QC). The same arrays were used for the second project, the “Northwestern NUgene Project”. Here, 1,239 individuals with 495,588 from the Human660W-Quad v1 array were available after QC, and, 267 individuals with 908,692 markers were available for Human1M-Duo v3. Finally the third project “GENEVA Diabetes Study”, comprised two further studies, the Nurses Health Study (NHS) and the Health Professionals Follow-up Study (HPFS). After provided quality control (QC), 3,435 individuals and 764,679 SNPs were available for NHS, and 2,606 individuals and 787,213 SNPs were available for HPFS. Both studies were performed on the Affymetrix^®^ Human SNP Array 6.0. In total, data from three different platforms with different marker content were used in six GWAS studies.

The dbGaP data was analyzed with naïve MA (MA-intersection), i.e., conventional MA restricted to the joint marker panel, with the proxy algorithm (MA-proxy), and based on data imputed with IMPUTEv2 [16], using 1,000 Genomes reference data. We relied on the QC data available from dbGaP, since our focus was on the relative performance of the various MA approaches rather than on the detection of novel associations.

Only 141,105 SNPs were available in all six studies and 561,282 SNPs were available in at least four studies. The proxy approach enabled the analysis of 1,427,514 SNPs and SNP/SNP–proxy combinations, whereof 1,0464,482 were available in all six studies and 1,279,702 were available in at least four studies. More than 85% of the proxies had an *r*^2^ greater than 0.8 with the substituted SNP.

Our philosophy was to compare the performance of the methods on Type II Diabetes genes that the community considers to be “undoubtedly” confirmed. To this purpose, we used the catalog of published GWAS results provided by the American National Human Genome Research Institute [1]. The catalog lists 33 Type II Diabetes GWAS genes/LD regions (“gene regions”) with at least one SNP that meets the genome-wide significance criterion of 5×10^−8^. For each of these gene regions, we investigated the 100 kb up- and downstream region of the SNP reported to be most significant and computed *minP*_*MA*−*impute*_ and *minP*_*MA*−*proxy*_ for the six studies available for us. Of note, a considerable part of the 33 genes was identified by meta-analysis efforts and shows only moderate odds ratios [24,25]. As a consequence, it cannot be expected that all the genes show measurable association effects within the smaller data sets we analyzed. In other words, not all the genes will be informative for the evaluation of the performance of the two meta-analysis approaches. Therefore, we restricted our comparison to gene regions that reached a significance level of 0.05 with at least one method.

In Table 4, 22 such gene regions are shown, together with the minimum p-values for all approaches. For 16 of the gene regions, MA-proxy yields a more significant result than MA-intersection. For TCF7L2 for instance, *minP* improves from 3.2×10^−19^ to 1.5×10^−22^, a change by a factor of 2127. In total, there are 6 gene regions with a p-value improvement of at least a factor of 10, including FTO, IRS1, JAZF1, KCNJ11, and KCNQ1. For KCNQ1, we observe *p* = 0.021 with MA-intersection and *p* = 0.00027 with MA-proxy,

an increase in significance by a factor of 78.6. For another 11 genes we observe an improvement with the proxy algorithm by a factor ranging from 2.18 (TSPAN8/LGR5) to 8.46 (JAZF1). There are also 5 gene regions for which no difference between MA-intersection and MA-proxy can be observed. In these cases, the most significant SNP is available in all 6 studies and, therefore, MA-intersection and MA-proxy coincide. Finally, for TSPAN8/LGR5, significance slightly decreases from 0.012 to 0.008. In summary, MA-proxy outperforms MA-intersection in the majority of cases and we observe an average (median) improvement of the level of significance of 107.1 (3.33), demonstrating the usefulness of proxy-SNPs.

**Table 4:** Comparison of point-wise and proxy MA of dBGaP GWAS for known type II diabetes genes

**PubMed ID**	**Gene**^**a**^	**rs-Catalogue**^**b**^	**p-Catalogue**^**c**^	**rs-Naive**^**d**^	**p-Naive**^**e**^	**rs-Proxy**^**f**^	**p-Proxy**^**g**^	**p-Impute**^**h**^	**Q(Na/Pro)**^**i**^	**Q(Pro/Imp)**^**j**^
20581827	BCL11A	rs243021	3.0E-15	rs243021	3.3E-03	rs11697597	3.3E-03	3.2E-03	1	1.03
20818381	C2CD4A,C2CD4B	rs7172432	9.0E-14	rs335302	1.2E-03	rs7172432	1.2E-03	1.98E-04	1	6.04
20581827	CDKAL1	rs10440833	2.0E-22	rs12336110	2.1E-03	rs6950237	2.1E-03	1.0E-03	1	2.10
19401414	CDKN2A, CDKN2B	rs2383208	2.0E-29	rs2383208	2.2E-03	rs2383208	8.4E-04	5.2E-04	2.6	1.60
20581827	CENTD2	rs1552224	1.0E-22	rs1552224	1.1E-01	rs1552224	3.4E-02	4.8E-03	3.33	7.08
17463249	FTO	rs8050136	7.0E-14	rs8050136	8.0E-03	rs8050136	6.7E-04	9.4E-04	11.9	0.71
20581827	HHEX,IDE	rs5015480	1.0E-15	rs5015480	9.6E-03	rs5015480	2.0E-03	4.9E-04	4.8	4.10
20581827	HMGA2	rs1531343	4.0E-09	rs12741948	3.3E-02	rs1122590	1.4E-02	9.1E-05	2.43	149.8
17463249	IGF2BP2	rs4402960	9.0E-16	rs4402960	2.3E-03	rs4402960	7.2E-04	1.2E-04	3.21	6.12
20581827	IRS1	rs7578326	5.0E-20	rs7578326	4.2E-02	rs7578326	1.7E-03	8.8E-04	24.65	1.95
18372903	JAZF1	rs864745	5.0E-14	rs864745	1.6E-03	rs864745	1.9E-04	1.1E-04	8.47	1.78
17463249	KCNJ11	rs5215	5.0E-11	rs5215	8.1E-02	rs4646410	3.1E-03	9.6E-04	26.16	3.24
18711367	KCNQ1	rs2237892	2.0E-42	rs2237892	2.1E-02	rs2237892	2.7E-04	3.5E-04	78.57	0.77
19734900	LOC64673, IRS1	rs2943641	9.0E-12	rs2943641	7.8E-02	rs2943641	1.7E-03	8.8E-04	45.94	1.95
20418489	RBMS1, ITGB6	rs7593730	4.0E-08	rs7593730	1.8E-05	rs7593730	3.4E-06	3.6E-06	5.15	0.94
20581827	SLC30A8	rs3802177	1.0E-08	rs2466295	2.4E-02	rs2466295	1.0E-02	5.3E-05	2.35	193.6
20862305	SPRY2	rs1359790	6.0E-09	rs17249026	4.5E-02	rs17249026	4.5E-02	2.1E-03	1	21.6
19734900	TCF7L2	rs7903146	1.0E-30	rs7903146	3.2E-19	rs7903146	1.5E-22	4.4E-23	2126.7	3.40
18372903	THADA	rs7578597	1.0E-09	rs2236705	1.5E-02	rs7578597	6.7E-03	2.4E-03	2.18	2.79
18372903	TSPAN8,LGR5	rs7961581	1.0E-09	rs4581087	1.1E-02	rs4581087	1.2E-02	2.4E-03	0.70	4.83
19734900	WFS1, PPP2R2C	rs4689388	1.0E-08	rs4689388	5.5E-03	rs4689388	1.6E-03	7.7E-04	3.44	2.08
20581827	ZFAND6	rs11634397	2.0E-09	rs11634397	2.6E-02	rs11634397	2.6E-02	1.0E-02	1	2.46

^a^Each gene region is listed only once, even if listed several times in the GWAS catalogue.

^b^Most significant SNP according to GWAS catalog [1].

^c^p-value according to GWAS catalog.

^d^Most significant SNP with naïve MA on intersection of marker panels of 6 dbGaP GWAS described before.

^e^p-value refering to SNP from previous column.

^f^Most significant SNP with proxy MA on 6 dbGaP GWAS.

^g^p-value refering to SNP from previous column.

^h^p-value of the correslonding SNP calculated by imputation/snptest.

^i^Improvement with proxy algorithm: quotient of columns “p-Pointwise” and “p-Proxy”.

^j^Improvement with imputation: quotient of columns “p-Proxy” and “p-Impute”.

Imputation-based MA outperforms MA-proxy in 19 out of 22 cases and we observe an average (median) improvement of the level of significance of 19.1 (2.79). In two cases, imputing outperforms the proxy-algorithm by a factor of more than 100, SLC30A8 (193) and HMGA2 (149.8), and in another case by a factor of 21.7 (SPRY2) which demonstrates the usefulness of long-range LD for association analysis. For 16 genes, the loss of significance with the proxy-algorithm is moderate with a factor of less than 10, of which for 11 genes we observe a factor of less than 4. For three genes, FTO, KCNQ1 and RBSM1/ITGB6, the proxy-algorithm even performs slightly better than MA-impute. In summary, one can say that the proxy algorithm yields good approximations of the actual level of significance in the majority of cases and that it is a potentially useful screening algorithm.

Table 5 contains a detailed example that explains the idea of proxy-SNPs for rs7903146 which is located within the gene of transcription factor TCF7L2, which has an essential function in the Wnt signaling pathway. This SNP is described in several GWAS to be significantly associated with the risk of Type II Diabetes. P-values up to 2×10^−51^ are reported [25]. In our dbGaP analysis, rs7903146(A/G) is present in studies 1-4, but not in studies 5 and 6. However, those studies contain rs4506565(T/A) which, according to 1,000 Genomes [19] data, has an *r*^2^ of 0.945 with rs7903146 and which is located only 2.3 kb downstream. Moreover, the A-T haplotype has a frequency of 0.668, while under linkage equilibrium a frequency of only 0.454 would be expected, based on the allele frequencies of 0.68 for rs7903146-A and 0.668 for rs4506565-T. Thus, rs7903146-A and rs4506565-T are “in-phase” alleles and proxy meta-analysis can combine the respective effect estimates and standard errors. Since in all 6 studies the effect estimates for rs7903146-A or rs4506565-T, respectively, are positive, joint meta-analysis becomes highly significant with a p-value of 1.5×10^−22^. Of course, rs7903146 also is indicated by conventional meta-analysis with a p-value of 3.3×10^−19^, but in this case only 4 GWAS studies can be used and the additional evidence coming from studies 5 and 6 is lost. We note that the proxy-SNPs that YAMAS uses may differ from study to study. In the example, the same proxy SNP was used for both study 5 and 6. In general, however, the proxy algorithm identifies for each study independently the SNP with the highest *r*^2^ with the SNP that shall be substituted.

**Table 5:** Proxy-Analysis of rs7903146 (TCF7L2)

**Study**^**a**^	**SNP/proxy-SNP**	**Chr**	**Position**	**EA**^**b**^	**OA**^**c**^	***β***^**d**^	**se**^**e**^	**P**^**f**^	**LD**^**g**^
1	rs7903146	10	114758349	A	G	0.39	0.09	1.5×10^−05^	-
2	rs7903146	10	114758349	A	G	0.68	0.20	7.0×10^−04^	-
3	rs7903146	10	114758349	A	G	0.40	0.13	3.0×10^−03^	-
4	rs7903146	10	114758349	A	G	0.44	0.08	1.6×10^−07^	-
5	rs4506565	10	114756041	T	A	0.20	0.05	2.0×10^−04^	0.945
6	rs4506565	10	114756041	T	A	0.30	0.06	3.8×10^−04^	0.945
Meta-Analysis	rs7903146	10	114758349	A	G	0.31	0.03	1.5×10^−22^	-

^a^Enumeration according to Table 1.

^b^Effect allele: the allele beta is given for.

^c^Other allele.

^d^Effect estimate according to logistic regression.

^e^Standard error.

^f^p-value.

^g^between SNP and proxy-SNP according to reference data (1,000 Genomes).

## Conclusion

Via real data analysis we were able to show that the proxy algorithm is not only fast and quickly employed, but also powerful. It clearly outperformed naïve SNP-by-SNP meta-analysis on real genotype data, when applied to a set of established Type 2 Diabetes regions. Moreover, our simulation study indicates that the proxy algorithm is very robust in terms of power with respect to a ethnically poorly matched reference panel. Thus, it is worth considering it as an alternative MA approach for studies on ethnical groups that are not directly represented in the 1,000 Genomes, for instance studies carried out in population isolates.

It is a known phenomenon that the catalogue of confirmed GWAS findings [1] is strikingly sparse for the X chromosome. At the moment, it is unclear if this phenomenon reflects the “true genetics” of human diseases or whether it is a detection bias. One might indeed speculate that the X chromosome is often ignored in MA efforts since it requires additional efforts to be imputed [26]. In this context, YAMAS may be particular helpful since no special analysis steps are necessary for the X chromosome.

Another notable feature of YAMAS is that it can be combined with imputed data. In practice, it may happen that studies are imputed with different reference panels. Moreover, particular SNPs might pass imputing-QC in some studies but not in others. In these situations, the analysis panel can be completed with the proxy-algorithm. In this context we wish to emphasize that our goal is not to compete with imputing as the standard approach for meta-analysis. Indeed, our own simulation study demonstrates a power advantage of imputation-based MA in a standard setting. Our aim is rather to speed up and give impetus to meta-analysis efforts. Even though the required analysis time for genome-wide imputing is meanwhile limited to a few weeks for experienced and well-equipped groups, joint projects are frequently long-lasting. Everyone who has worked in a meta-analysis project has either experienced or can imagine that it can easily take one year until all participating groups have provided their imputing results, either because some of the participating groups are less experienced in imputing analysis than others or either because they are involved in projects which they assign higher priority to. In particular, the priorities the participants have will sometimes be heterogeneous, causing delay for those who have the primary interest in the joint effort. Therefore, we believe that a method that facilitates MA and yields ad hoc, but still interpretable and meaningful results, is highly warranted. The proxy algorithm we have introduced fulfills these criteria: it directly operates on GWAS analysis results and can be run in a few hours even when the meta-analysis comprises many groups, and has descent power since it can analyze all SNPs that were genotyped in at least one of the participating studies.

## Methods

### Standard Meta-Analysis

YAMAS either combines allelic odds ratios (*ORs*) or estimates of the allelic effects (“betas”) as obtained in regression models. In case of *ORs*, the effect becomes *E*=*ln*(*OR*), else it stays un-transformed. Effects are merged across studies with the weighted average,

(1) $$Ē=\frac{\overset{k}{\underset{i=1}{\sum }}w_{i}\cdot E_{i}}{\overset{k}{\underset{i=1}{\sum }}w_{i}}$$

, where *k* is the number of studies and *w*_*i*_ is a weight given by the standard error (*SE*_*i*_) for the *i*^*th*^ study: $w_{i}={\left(SE_{i}^{2}\right)}^{-1}$. The standard error of *Ē* is computed as

(2) $${\text{SE}}_{Ē}=\sqrt{\frac{1}{\overset{k}{\underset{i=1}{\sum }}w_{i}}}$$

and the combined two-tailed p-value becomes $p=2\cdot (1-\Lambda (|Ē/SE_{Ē}|))$, with $\Lambda (|Ē/SE_{Ē}|)$ being the standard normal cumulative density distribution function. The meta-analysis follows the standards exemplified [8] and is also equipped to consider the between-study variance of markers by calculating so called random effect sizes [27,28]. Taking Cochran’s *Q*-value [29],

(3) $$Q=\overset{k}{\underset{i=1}{\sum }}w_{i}{\left(E_{i}-Ē\right)}^{2}\;,$$

, as an indicator for the total between-study variance, we are able to replace *w* with *w*^∗^ = (*SE*^2^ + *τ*^2^)^−1^ and use this weight to reflect heterogeneity, *τ*^2^ being the between-study variance:

(4) $$\tau^{2}=\frac{Q-\text{df}}{(\overset{k}{\underset{i=1}{\sum }}w_{i}/\overset{k}{\underset{i=1}{\sum }}w_{i}E_{i}^{2}})$$

if *Q*−(*k*−1) > 0, else *τ*^2^ = 0. *Q* itself follows a *χ*^2^-distribution with *k* − 1 degrees of freedom [30]. We note that it has recently been shown [22] that the classical random-effects model tests a too strict null hypothesis and although intended for effects that vary between studies, ironically enough leads to a conservative procedure in the presence of heterogeneity. However, since the random-effects model is still often requested by reviewers, we still feature it in our software.

### The Proxy Algorithm

**Figure 4:**
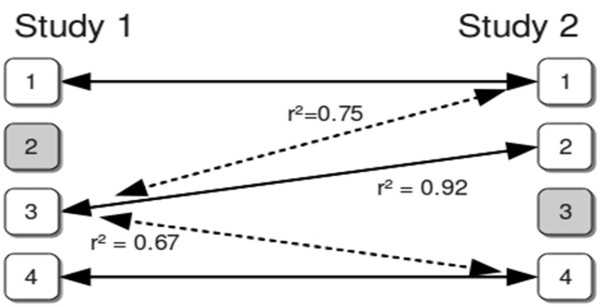
**Proxy meta-analysis schematic example.** Schematic example of a meta-analysis with proxy markers. For simplicity we consider only two studies with four markers each (1-4). Common MA is applied on markers 1 and 4 (as they are present in both marker sets), yet when YAMAS hits marker 3, which is missing in the second study (3 – gray box), it selects marker 2 in study 2 as its proxy marker, based on the *r*^2^ indicator. Dashed arrows indicate non-chosen potential proxy markers. The case of the missing marker 2 in study 1 is omitted for better readability.

We assume that association results (effect estimates and standard errors) are available for the **real** genotype data of each participating study. In order to enable MA on the complete marker panel missing markers can be filled with “proxy markers”. For this purpose, a sample reference file based on 1,000 Genomes [19] SNP content is provided for download on the YAMAS web site. As an alternative, own reference files can be produced using genotype data in PLINK-format [10] with the current version of INTERSNP [31]. The reference file tabulates pairs of SNPs with marker IDs, each marker’s alleles, the chromosome the markers are on, their absolute physical distance in base pairs, *r*^2^ as a linkage disequilibrium indicator [32] and a boolean flag to define the proxy alleles (see below). We provide proxy files for CEU, YRI and JPT+HCN samples. In general, pairs of SNPs no more than 200kb apart and with an *r*^2^ ≥ 0.5 are listed. For the X chromosome and the MHC region, we choose a distance limit of 5 Mb. If the algorithm would encounter a situation where one marker is present in one of the studies, but missing in one or more of the other studies, it will try to find a proxy marker in those studies, compare Figure 4. Proxy markers are ranked by their mutual *r*^2^ (higher *r*^2^ ranks higher). This sorted list of markers is tried for the presence in the data set. The first present SNP, i.e., the SNP with the highest LD with the missing SNP, is chosen to be the proxy-SNP and will subsequently be used for MA, see also Figure 4. To account for the effect direction the proxy marker also carries the information for a proxy allele: the reference file designates the allele as the proxy allele for which the observed haplotype frequency is greater than the expected haplotype frequency under linkage equilibrium. In other words, the “in-phase” alleles of a SNP and its proxy define mutual proxy alleles (cf. also the section “Analysis of dbGaP data” for an example). A boolean indicator for the proxy alleles is part of the reference file. In summary, meta-analysis is always based on the established formula

(5) $$Ē=\frac{\overset{k}{\underset{i=1}{\sum }}w_{i}\cdot E_{i}}{\overset{k}{\underset{i=1}{\sum }}w_{i}}\;,$$

from the previous section. In contrast to “standard” meta-analysis the effect estimates that are combined do not always refer to the same SNP (rsNumber) in each study, but to a SNP from one study and its proxy-SNPs in other studies. In order to select a SNP as a proxy, we require a minimum *r*^2^ of 0.50.

## URLs

YAMAS, Yet Another Meta-Analysis Software; http://yamas.meb.uni-bonn.de/ The OpenMP API specification for parallel programming, http://openmp.org/wp.

## Competing interests

The authors declare that they have no competing interests.

## Author’s contributions

CM and TB developed the proxy algorithm and are responsible for the software design. CM programmed the major part of YAMAS. ML and TB prepared the manuscript. ML is responsible for the programming of additional features and software maintenance. MA performed parts of the T2D analysis. CH, MM, DD and AL provided significant input to the work and the manuscript. All authors read and approved the final manuscript.
